# DiagPat: An Explainable Language Detection Model Using EEG Signals

**DOI:** 10.3390/s26113363

**Published:** 2026-05-26

**Authors:** Tugce Keles, Kubra Yildirim, Dahiru Tanko, Suat Tas, Irem Tasci, Burak Tasci, Gulay Tasci, Turker Tuncer, Sengul Dogan

**Affiliations:** 1 Department of Digital Forensics Engineering, College of Technology, Firat University, 23119 Elazig, Turkey; tkeles@firat.edu.tr (T.K.); kubra.yildirim@firat.edu.tr (K.Y.); 231144106@firat.edu.tr (S.T.); turkertuncer@firat.edu.tr (T.T.); sdogan@firat.edu.tr (S.D.); 2 Department of Computer Engineering, Faculty of Engineering and Architecture, Erzurum Technical University, 25050 Erzurum, Turkey; dahiru.tanko@erzurum.edu.tr; 3 Department of Neurology, School of Medicine, Firat University, 23200 Elazig, Turkey; itasci@firat.edu.tr; 4 Vocational School of Technical Sciences, Firat University, 23119 Elazig, Turkey; 5 Department of Psychiatry, Elazig Fethi Sekin City Hospital, 23300 Elazig, Turkey

**Keywords:** DiagPat, EEG language detection, cognitive science, neuroscience, explainable feature engineering

## Abstract

**Highlights:**

- DiagPat classified Arabic–Turkish EEG tasks with 92.14–99.35% accuracy.
- DLob maps revealed case-specific frontal-dominant cortical transition patterns.
- Lightweight XFE models can support interpretable EEG language decoding.
- LOSO results show that subject-independent BCI use needs further validation.

**Abstract:**

Electroencephalography (EEG) offers a non-invasive and cost-effective means of probing brain activity during language processing; however, prior EEG-based language studies have been limited by small datasets, a predominant focus on native-speaker or speech-unit recognition rather than direct language detection, evaluation on only a small number of experimental settings, and frequent reliance on computationally intensive deep learning models with limited interpretability. The proposed feature engineering models classifies EEG segments by language and task mode. The languages are Arabic and Turkish. The modes are reading and listening. In this study, a signal refers to one fixed-length multi-channel EEG segment (14 channels × 15 s at 128 Hz). A channel refers to one electrode time series within that segment. To address these gaps, we curated a new EEG language detection dataset from 346 participants (98 Arabic and 248 Turkish) recorded in reading and listening modes, yielding 6364 EEG segments. Using this dataset, we proposed DiagPat, an explainable feature engineering (XFE) model that extracts transition table-based features from both EEG channels and signals through diagonal pattern analysis. The model combines DiagPat feature extraction with iterative neighborhood component analysis (INCA) for feature selection, at algorithm-based k-nearest neighbors (tkNN) classifier for prediction, and the Directed Lobish (DLob) symbolic language for explainability. We evaluated the framework across nine classification cases covering language detection, mode detection, and mixed multi-class settings. The proposed DiagPat-driven XFE model achieved more than 90% accuracy in all cases, with accuracies ranging from 92.14% to 99.35%, and generated case-specific cortical connectome diagrams that supported the interpretable characterization of language- and mode-related brain activity. Subject-independent results were also reported using leave-one-subject-out cross-validation (LOSO CV), where LOSO accuracies ranged from 29.75% to 83.50%. Thus, the 10-fold CV results show segment-level performance, whereas the LOSO results show subject-level generalization. Balanced accuracy and macro-F1 are also reported. These findings indicate that DiagPat provides an accurate, lightweight, and explainable framework for EEG-based language detection.

## 1. Introduction

Electroencephalography (EEG) is a tool used to monitor the electrical activity of the brain [1,2]. EEG records electrical potentials in brain regions through electrodes placed on the scalp in a specific order. This method is cost-effective and non-invasive [3]. The EEG background is kept brief. Only the information needed for this study is provided.

EEG was initially developed for the detection and treatment of neurological diseases [4]. However, with technological advancements, it has become possible to obtain EEG data using portable devices [5], enabling the use of EEG signals not only in medicine but also in various other disciplines [6]. EEG signals, which are generally used in diagnosing sleep disorders, epilepsy, and analyzing auditory states, have become an important tool for areas such as stress management and the monitoring of learning processes [7,8,9].

In recent years, the use of brain–computer interfaces (BCIs) has rapidly developed, and with this, the application of EEG in areas such as language perception and processing is also increasing [10]. These developments offer innovative solutions, especially for individuals who have lost the ability to speak. In this way, individuals who have lost their ability to speak can express their thoughts through alternative methods [11,12]. This improves their quality of life and supports their continued engagement with the world [13].

Language, the cornerstone of human communication, plays an important role in transferring emotions, thoughts, and cultural values [14]. During language perception, electrical activity is produced by the human brain [15]. This electrical activity provides an important source for understanding the neurological characteristics of language [16]. EEG signals have made it possible to study the brain’s mechanisms during language processing. This creates opportunities in fields such as language learning and language therapy [17].

In this study, a new model that aims to detect language using EEG signals is presented. Texts in Arabic and Turkish were provided to participants in reading and listening modes for this purpose. The DiagPat model proposed in the study is an explainable feature engineering model. The model was tested in nine different cases, and its performance was analyzed. This study highlights the potential of language detection using EEG signals and paves the way for future research.

Nine classification cases were defined with the curated dataset. Case 1 separates Arabic reading from Arabic listening. Case 2 separates Arabic reading from Turkish reading. Case 3 separates Arabic reading from Turkish listening. Case 4 separates Arabic listening from Turkish reading. Case 5 separates Arabic listening from Turkish listening. Case 6 separates Turkish reading from Turkish listening. Case 7 detects mode (reading vs. listening) in the full dataset. Case 8 is a four-class task that includes all classes. Case 9 detects language (Arabic vs. Turkish) in the full dataset. DiagPat was used for feature extraction. iterative neighborhood component analysis (INCA) was used for feature selection. tkNN was used for classification. DLob was used for explainability.

### 1.1. Related Works

Tuncer et al. [18] introduced a novel channel pattern (ChannelPat) and the Lobish symbolic language in their study. To enhance performance, they employed INCA and a new ensemble k-nearest neighbor (tkNN) classifier. The study utilized a newly collected EEG dataset comprising 20 daily life sentences in Arabic and Turkish. Data were recorded from 75 participants, with the Lobish language being applied to achieve explainable results. The model achieved a classification accuracy of 98.59%. However, the dataset was limited, and the Lobish language only represented the four lobes of the brain. Barua et al. [19] developed an automatic sentence classification model based on EEG signals. They created a dataset involving 40 participants exposed to 20 standardized sentences in their native languages, English and Turkish, through display and listening modes. Using the dynamic dimensional binary pattern (DSBP) feature extractor and the iterative multiple classifiers-based majority voting (IMCMV) algorithm, they achieved classification accuracies of 98.81% in display mode and 98.19% in listening mode. Despite these results, the dataset’s limited scope and lack of testing on alternative datasets posed challenges to the model’s generalizability. Bakshali et al. [20] classified imagined speech commands through EEG signals by analyzing Correntropy Spectral Density matrices and extracting features using Riemannian geometry. The study utilized the Kara One dataset, which included data from eight participants. The proposed method achieved a classification accuracy of 90.25%, although accuracy was lower for words with similar pronunciations, and the dataset’s limited size restricted generalization. Keles et al. [21] proposed a model for EEG-based sentence classification by creating three new datasets of Turkish and English sentences. These included listening-based and demonstrative datasets. They used the TesPat textural feature generator for feature extraction and neighboring component analysis for feature selection. The model achieved accuracy rates exceeding 97% for cross-validation (CV) and over 91% for leave-one-subject-out cross-validation (LOSO CV). However, the limited number of participants, sentences, and languages constrained the model’s generalizability. Sarmiento et al. [22] explored speech command recognition using EEG signals. Their study focused on Spanish vowels (a, e, i, o, u) and utilized two datasets: BD1, comprising imagined vowels from 15 participants, and BD2, containing EEG signals from the left hemisphere areas of 50 participants. A new deep learning algorithm, CNNeeg1-1, achieved accuracy rates of 65.62% for BD1 and 85.66% for BD2. While the larger, artifact-controlled BD2 dataset improved accuracy, the study’s reliance on a limited number of electrodes and brain regions posed limitations. Vorontsova et al. [23] aimed to develop a silent speech recognition system using EEG signals. They focused on eight Russian words and a pseudo-word for control purposes, with data collected from 268 healthy participants. Using a ResNet18 + 2GRU model, they achieved an accuracy of 84.5%. However, the study’s scope was restricted to Slavic languages and a small vocabulary, limiting the model’s applicability. Datta et al. [24] classified imagined words into nouns and verbs using EEG signals. Words included five nouns (e.g., apple, laptop) and five verbs (e.g., run, swim). Spectrograms obtained from the EEG signals were classified using the MC-CNN architecture, achieving a 78.5% accuracy rate with LOSO validation. However, the small participant pool and limited vocabulary size restricted the model’s generalization. Lingwei et al. [25] proposed a method for detecting imagined speech using an open EEG dataset from UNER, involving 15 participants and words such as arriba, abajo, and izquierda. The discrete wavelet transform (DWT) was used for feature extraction, and a support vector machines (SVM) were applied for classification. The model achieved a 79.78% accuracy rate for individual participant classification but only 26.75% for mixed-participant classification, highlighting its limitations in general model creation. Puffay et al. [26] investigated speech perception using EEG signals, revealing perceptual differences between native and foreign languages. The study exposed 26 native Dutch speakers to Dutch, Frisian, and mixed Dutch text, achieving a 71% accuracy rate for Frisian and mixed Dutch using a CNN-based model. However, the study’s limited language diversity hindered generalization across languages. Alonso-Vazquez et al. [27] classified words using EEG signals with data from 28 healthy participants aged 20 to 47. Comparing two methods, PSD+SVM and EEGNet, the study found that EEGNet performed better in three out of four scenarios. While promising, the study was restricted to healthy individuals and lacked sufficient accuracy for online applications.

A summary of the related works is given in Table 1. The table reports the model, dataset/task, languages, number of subjects, and accuracies, enabling a clearer comparison across studies.

### 1.2. State-of-the-Art Limitations

- The most commonly used EEG datasets are relatively small. A large EEG signal dataset should be collected.
- In the literature, most research focuses on native speaker detection [28,29]. There is limited research on language detection.
- Most studies have use only one case to demonstrate success [30], which limits the ability to show the general classification performance of a model.
- There are various EEG classification models in the literature, and most of them focus only on attaining high classification performance [31]. To achieve high classification performance, most researchers also use deep learning models [32,33]. However, the time complexity of deep learning models is exponential.

### 1.3. Motivation and Our Method

Our main motivation is to investigate brain-based language detection. Therefore, a new EEG language detection dataset was collected. By deploying this dataset, nine cases were created. By collecting this dataset, this research filled the first identified literature gap.

To address the second identified literature gap, we collected the language detection dataset from 346 participants in two modes: (i) reading and (ii) listening.

By utilizing the collected dataset, nine cases were defined, and we tested the recommended DiagPat-driven explainable feature engineering (XFE) model on these nine cases.

As stated in the literature gap 4, we presented a new XFE model, which consists of four major phases: (i) feature extraction using the recommended DiagPat, (ii) feature selection using iterative neighborhood feature selection (INCA) [34], (iii) classification results generation applying a t algorithm-based k-nearest neighbors (tkNN) [18], and (iv) DLob [35]-based interpretable results generation.

In this model, features were extracted by deploying the proposed DiagPat. To choose the most informative features, the INCA feature selector was utilized. The selected features were used for interpretable results generation and classification results generation. The selected features were provided as input to the tkNN classifier, and the classification results were obtained. By utilizing the identities of the selected features, the DLob sentence was generated. Using the created DLob sentence, a cortical connectome diagram for each case was created.

### 1.4. Innovations and Contributions

Novelties:

- In this research, a new EEG language detection dataset was curated. By using the curated dataset, nine different cases were defined.
- A new generation feature extraction function was presented, and this feature extraction function was named the DiagPat feature extraction function.

Contributions:

- The curated EEG language detection dataset is larger than the previously collected EEG language detection datasets. By using this dataset, we defined nine cases, and each case represented a unique situation related to language detection. In this aspect, by collecting this dataset, we contributed to the brain-based language detection research area.
- By proposing the DiagPat feature extraction function, we extracted features from both channels and signals. Moreover, a new XFE model was presented. The introduced DiagPat-driven XFE model attained over 90% classification accuracy for all nine defined cases. In this respect, the introduced model contributes to EEG signal classification and feature engineering.
- This research contributes to explainable artificial intelligence (XAI) since DLob has been integrated into the DiagPat-based XFE model, generating interpretable results. By deploying DLob, we extracted connectome diagrams related to language detection. In this respect, the presented DiagPat-driven XFE also contributes to neuroscience.

## 2. Language Detection Dataset

To curate this dataset, we used participants with two nationalities: (i) Arabic and (ii) Turkish. There were 98 Arabic and 248 Turkish participants. In order to collect EEG signals from these participants, we used a paragraph from the Alchemic classic. In the reading mode, the participants read the given paragraph, and in the listening mode, participants listened to this paragraph in their language. The English version of the used paragraph is “*The boy’s name was Santiago…*” paragraph.

To collect the EEG signals from these participants, the Emotiv Epoch X brain cap was used. This brain cap has 14 channels, and the sampling rate is 128 Hz. In order to create EEG segments, the EEG signals were divided into 15 s segments. In this respect, 6364 EEG segments were obtained from the collected EEG signal dataset. The features of the collected EEG signal dataset are presented in Table 2.

Experimental protocol. The same paragraph from The Alchemist was used in both languages. The Arabic and Turkish versions were professional translations of the same source text. Text length was matched as closely as translation allowed. In the reading mode, participants read the paragraph silently at their own pace. In the listening mode, participants listened to the paragraph in their own language through closed headphones. The audio was recorded by one trained native speaker for each language. Both recordings were made in the same quiet room with the same microphone and setup. The sampling rate was 44.1 kHz. Loudness was normalized. Each audio file lasted about three minutes. Each session started with a 30 s eyes-open rest. The task then started. A one-minute rest was given between the reading and listening tasks. The task order was counterbalanced across participants. Room lighting was kept constant. Participants kept their eyes open during reading and closed during listening. Long EEG recordings were divided into non-overlapping 15 s segments.

EEG preprocessing. The raw EEG was first re-referenced to the common average. A fourth-order Butterworth band-pass filter was applied from 0.5 to 45 Hz. A 50 Hz notch filter was applied to reduce power-line noise. Bad channels were identified when their standard deviation was much lower or higher than that of the other channels. These channels were interpolated from neighboring channels. Eye-blink, muscle, and cardiac artifacts were reduced by independent component analysis (ICA). Components with clear artifact patterns were removed. The cleaned signal was then divided into non-overlapping 15 s segments. Preprocessing was fitted only on the training fold and then applied to the test fold. Thus, no test-fold information was used to estimate the filters or ICA. This rule was applied to both 10-fold CV and LOSO CV.

## 3. Diagonal Pattern

The major objective of the presented feature extraction function is to extract meaningful features to detect differences between channels and signals. To achieve this aim, an overlapping block division was applied, and square matrices were created. By using the diagonal values of these matrices, the features were extracted. The steps of this feature extraction procedure are:

The terms “channel” and “signal” are used as follows. A channel is one electrode time series. The dataset contains 14 channels per recording. A signal is a multi-channel matrix of one EEG segment. Thus, one signal is a 14 × N matrix, where N is the number of time samples. DiagPat takes one signal as input. The transition table is built across the 14 channels of that signal. The resulting features describe how the channel order changes over time within the same signal.

S1: Define the 3D transition table and fill all elements with zero.

(1) tt=zeros(ch,ch,5)

Herein, tt: transition table, zeros(.,.,.): zero filling function and ch: number of channels. In this research, the used dataset has 14 channels. Therefore, the length of the transition table is 14 × 14 × 5.

S2: Generate matrix with a size of ch×ch from the used signal.

(2) $$mat=signal\left(k+i,j\right),\;i\in \left\{1,2,\ldots ,ch\right\},j\in \left\{1,2,\ldots ,ch\right\},k\in \{1,2,\ldots ,L-ch+1\}$$

where mat: the generated matrix and L: length of the signal.

S3: Sort each row of the generated matrix and create the transformed matrix.

(3) $$idx=argsort\left(-mat\left(i,:\right)\right)$$

(4) trm(i,:)=idx

Herein, idx: the sorted indices and trm: transformed matrix.

S4: Store diagonal and inverse diagonal values of the transformed matrix.

(5) $$d\left(j,1\right)=trm(i,i)$$

(6) $$d\left(j,2\right)=trm(i,ch+1-i)$$

where d: diagonal and inverse diagonal values of the transformed matrix.

S5: Fill the transition table by deploying

(7) $$tt\left(d\left(h,1\right),d\left(h+1,1\right),1\right)+=1,h\;\in \left\{1,2,\ldots ,ch-1\right\}$$

(8) $$tt\left(d\left(h,2\right),d\left(h+1,2\right),2\right)+=1$$

(9) $$tt\left(d\left(h,1\right),d\left(h+1,2\right),3\right)+=1$$

(10) $$tt\left(d\left(h,2\right),d\left(h+1,1\right),4\right)+=1$$

(11) $$tt\left(d\left(i,1\right),d\left(i,1\right),5\right)+=1$$

Here, we have computed the transition table of the used matrix.

S6: Repeat S1–S5 until the total number of matrices of the signal is reached.

S7: Apply matrix-to-vector transformation to obtain feature vector.

(12) $$vec\left(q\right)=tt\left(a,b,c\right),\;\left(a,b\right)\in \left\{1,2,\ldots ,ch\right\},\;c\in ,\;q\in \left\{1,2,\ldots ,5ch^{2}\right\}$$

where vec: feature vector with a length of $5ch^{2}$.

The given six steps (S1–S7) define the presented DiagPat and the recommended DiagPat.

## 4. The Presented DiagPat-Based Explainable Feature Engineering

**Figure 1:**
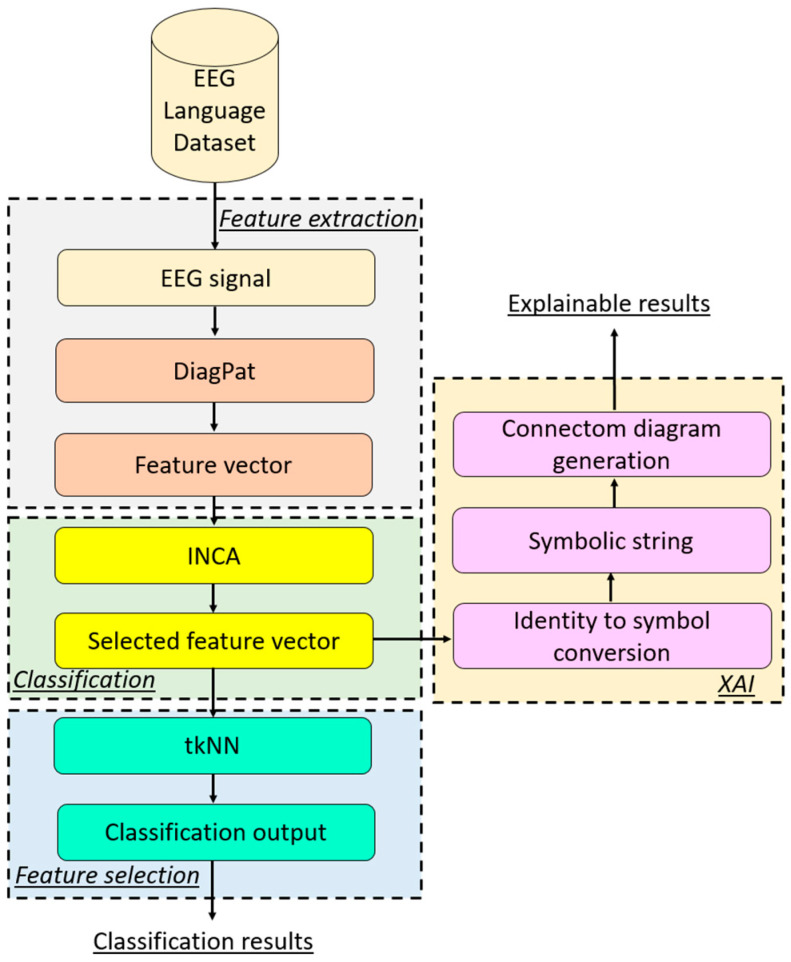
The graphical illustration of the recommended DiagPat-based XFE model.

In this research, we aimed to achieve high classification accuracy with linear time complexity. Moreover, explainable results were generated by deploying the DLob symbolic language. To achieve high classification performance, we used two self-organized methods: the INCA and the tkNN classifiers. In the feature extraction phase, DiagPat extracted features from signals. In this research, the EEG signal dataset used has 14 channels. Therefore, the presented DiagPat produces 960 (=14 × 14 × 5) features. The most informative of these features were chosen by the INCA feature selector. The selected features were utilized as input for the tkNN classifier with 10-fold cross-validation. To generate interpretable results, DLob was used, and cortical connectome diagrams for the cases were obtained. The illustration of the presented DiagPat-driven model is also demonstrated in Figure 1.

To give more details about the recommended model, the steps of this model are:

Phase 1 (Feature extraction): Extract features by deploying the introduced DiagPat feature extraction function.

(13) $$X\left(d,:\right)=DP\left(signal_{d}\right),\;d\in \{1,2,\ldots ,Dim\}$$

Herein, X is the feature matrix, DP(.) is the presented DiagPat feature extraction function and Dim is the number of the EEG signals.

Phase 2 (Feature selection): Choose the most informative features by deploying the INCA feature selector. The INCA feature selector is an iterative and self-organized feature selection function.

(14) $$[SX,idx]=INCA\left(X,y\right)$$

Herein, SX is the selected feature vector, idx represents the identities of the selected features, $INCA\left(.\right)$ is the INCA feature selection function and y represents the actual labels.

Phase 3 (Classification): Classify the selected features by deploying tkNN classifier. The tkNN classifier is an ensemble and self-organized classifier that was introduced by Tuncer et al. [18,35] in 2024. By deploying the tkNN classifier, the classification outcome was obtained.

(15) $$out=tkNN\left(SX,y\right)$$

Herein, out is the selected feature vector, tkNN(.) is he tkNN classification function and y represents actual labels.

Phase 4 (XAI): Generate interpretable results by deploying DLob [35] symbolic language.

(16) $$snt=DLob\left(idx\right)$$

where snt is the extracted DLob sentence and DLob(.) is the DLob sentence creation function.

Deploying these four phases, both classification and explainable results were generated.

## 5. Experimental Results

The results of the presented model have been presented in this section.

### 5.1. Experimental Settings

The introduced DiagPat-driven model was implemented in the MATLAB (version 2024a) programming environment. We used a simple, configured computer, and the recommended DiagPat-driven model was implemented using central processing unit (CPU) mode since the model has linear time complexity. This model is a parametric EEG classification model, and the parameters used in this model are tabulated in Table 3.

By deploying these parameters, the recommended DiagPat-based model was implemented. This model was functional, and the functions used include (i) main, (ii) DiagPat, (iii) INCA, (iv) tkNN, and (v) XAI. These functions have been stored as m-files.

### 5.2. Time Complexity Analysis

The first evaluation metric is the time complexity analysis. The time complexity analysis of the introduced DiagPat-driven XFE model was computed using big O notation on a phase-by-phase basis, as detailed below.

Feature extraction: In the feature extraction phase, the introduced DiagPat feature extractor was used. The presented DiagPat is a simple and effective feature extractor, and the time complexity of this feature extraction phase is O(S), where S is the size of the EEG signal.

Feature selection: To choose the most informative features, the INCA feature selector was used. This feature selector is an iterative and self-organized feature selector. INCA’s time complexity is computed as O(N+RK+G), where N is the time complexity coefficient of the NCA, R is the range of the iteration, G is the time complexity of the greedy algorithm, and K is the time complexity of the kNN classifier.

Classification: The tkNN classifier was used to create classification results, and this classifier is an ensemble and a self-organized classifier. The time complexity of the tkNN classifier is O(PK+I+G), where P is the number of parameters and I is the time complexity of the IMV algorithm.

XAI: In the XAI phase, DLob has been used, leveraging the identities of the selected features. Therefore, the time burden of the XAI phase is O(C), where C is the number of chosen features.

Overall: The total complexity of the introduced DiagPat-driven model is computed as $O\left(S+N+RK+G+PK+I+G+C\right)≅\;O(S+N+R(K+P)+G+I+C)$, which is a linear time complexity. Therefore, the presented DiagPat-driven model is a lightweight model, and there is no need to use parallel programming or a multi-core processor.

### 5.3. Cases

To obtain both classification and interpretable results, we defined nine cases. The main objective of the presented cases is to clearly explain the brain’s state for language detection. The defined cases are explained below.

Case 1: Classification of the Arabic language EEG collection modes. In this case, there are 872 Arabic reading and 2125 Arabic listening EEG signals.

Case 2: This case is motivated by the classification of Arabic and Turkish languages in reading mode. There are 2085 EEG signals in this case (Arabic reading: 872, Turkish reading: 1273).

Case 3: In this case, Arabic reading (872 EEG signals) and Turkish listening (2094 EEG signals) classes have been used.

Case 4: Arabic listening (2125 EEG signals) and Turkish reading (1273 EEG signals) were used in this case.

Case 5: The main objective of this case is to detect the language in the listening mode.

Case 6: This case aims to detect the collection mode (reading or listening) using Turkish EEG signals.

Case 7: This case aims to detect the language used. Therefore, it groups Arabic listening and reading into one class, while the other class consists of Turkish listening and reading. In this case, all EEG signals in the curated dataset have been used, totaling 6364 EEG signals.

Case 8: In this case, there are four classes, and it uses all EEG signals and classes. A total of 6364 EEG signals have been used in this case.

Case 9: This case aims to detect the EEG collection mode. Therefore, the first class is reading (Arabic reading + Turkish reading), and the second class is listening (Arabic listening + Turkish listening). In this case, there are 6364 EEG signals.

By using these defined cases, we presented the classification and interpretable results.

### 5.4. Classification Results

**Figure 2:**
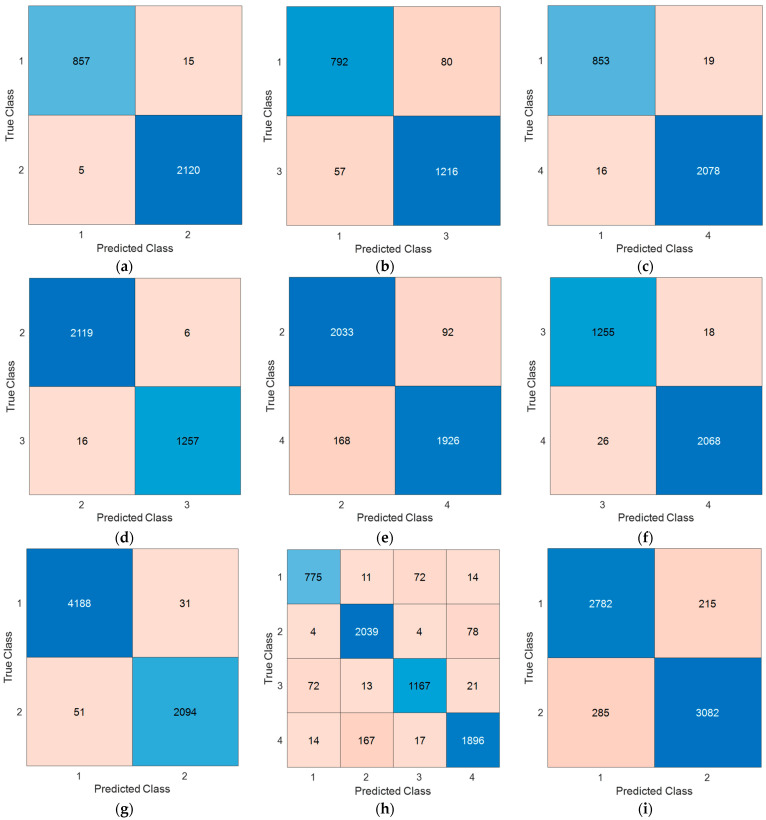
The computed confusion matrices of the cases. (**a**) Case 1. Herein, 1: Arabic reading, 2: Arabic listening. (**b**) Case 2. Herein, 1: Arabic reading, 2: Turkish reading. (**c**) Case 3. Here, 1: Arabic reading, 4: Turkish listening. (**d**) Case 4. Herein, 2: Arabic listening, 3: Turkish reading. (**e**) Case 5. Here, 2: Arabic listening, 4: Turkish listening. (**f**) Case 6. Herein, 3: Turkish reading, 4: Turkish listening. (**g**) Case 7. Here, 1: Reading, 2: Listening. (**h**) Case 8. Herein, 1: Arabic reading, 2: Arabic listening, 3: Turkish reading, 4: Turkish listening. (**i**) Case 9. Here, 1: Arabic, 2: Turkish.

To obtain classification results, the introduced DiagPat-driven XFE model was applied to the defined cases. In order to evaluate the classification performance of these nine cases, classification accuracy, precision, recall, F1-score, and geometric mean were used. To compute these metrics, the resulting confusion matrices were generated, as depicted in Figure 2.

According to the computed confusion matrices, the classification results are tabulated in Table 4.

The given results (see Table 4) clearly show that the introduced DiagPat-driven model achieved over 91% classification performance across all performance metrics. Moreover, we highlighted the best results using bold font. According to the results presented in Table 4, the most accurate case is Case 4, which consists of two classes: Arabic listening and Turkish reading. In this case, the languages and modes are different, which contributed to the best classification accuracy. Additionally, the language detection case (Case 9) achieved a classification accuracy of 92.14%, and the mode detection task (reading or listening, Case 7) yielded a classification accuracy of 98.71%. Furthermore, the four-class case (Case 8) achieved a classification accuracy of 92.35%. For these cases (from Case 1 to Case 9), the range of classification accuracies was between 92.14% and 99.35%. These results clearly demonstrate the general classification performance of the introduced DiagPat-driven XFE model.

### 5.5. Explainable Results

The introduced EEG classification model is an XFE model as it generates interpretable results using the DLob symbolic language. The DLob symbolic language is a new-generation XAI method that explains the brain’s state using symbols, with each symbol representing a cortical area and its direction.

In this research, our DiagPat-driven model was tested on nine cases. By utilizing the identities of the selected features, DLob symbols were extracted. By merging these extracted DLob symbols, a DLob string was created. The histogram, information entropy, and connectome diagrams were computed from the produced DLob sentences, and the interpretable results were generated.

The DLob sentences generated by the proposed DiagPat-based model are provided in the Supplementary Materials. Moreover, the computed information entropies of the generated DLob sentences are tabulated in Table 5.

**Figure 3:**
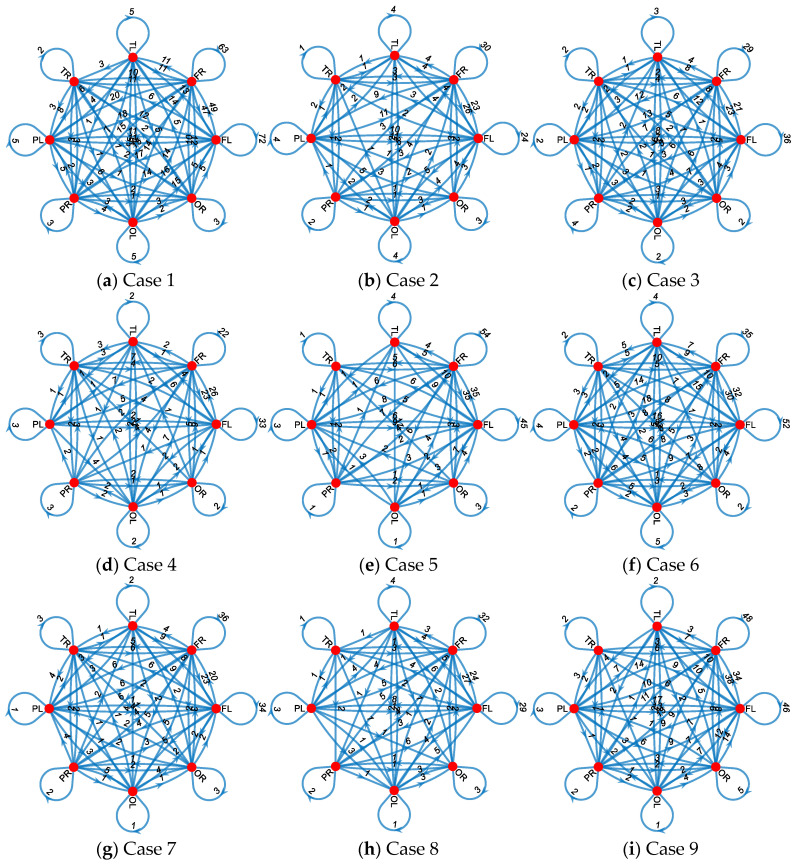
The generated connectome diagrams of the used cases. Herein, FL: Frontal Left, FR: Frontal Right, TL: Temporal Left, TR: Temporal Right, PL: Parietal Left, PR: Parietal Right, OL: Occipital Left, OR: Occipital Right.

Table 5 demonstrates that the computed DLob sentences are predictable. Therefore, brain-based language and mode detection is a predictable process. The most complex case is Case 6, with a complexity ratio of 89.33, which defines reading and listening discrimination. Moreover, the extracted cortical connectome diagrams for the defined nine cases are shown in Figure 3.

Figure 3 showcases that the introduced DiagPat-based model generates unique connectome diagrams for each case. These cortical connectome diagrams are used as feature vectors (which were generated using the transition table of the symbols). Furthermore, Figure 3 highlights the dominant activation of the frontal lobe.

## 6. Discussion

In this research, a new-generation EEG signal classification model was presented, termed the DiagPat-driven XFE model. Our model achieved over 92% classification accuracy for the defined nine cases. Moreover, the introduced DiagPat-driven XFE model attained over 99% classification accuracy for Case 1 and Case 3. These cases involve Arabic reading and listening discrimination, as well as Arabic reading and Turkish listening discrimination.

Segment-level vs. subject-independent results. EEG decoding is subject-dependent. In 10-fold CV, the evaluation is segment-level, and segments from the same participant may appear in both training and test folds. Thus, the model may learn participant-specific or session-specific patterns. This risk was tested with leave-one-subject-out cross-validation (LOSO CV). In LOSO CV, all segments from the test participant are excluded from training. The LOSO accuracies range from 29.75% to 83.50%. The gap between 10-fold CV and LOSO CV shows that part of the high 10-fold performance is related to subject-level information, not only language-related EEG activity. Therefore, both metrics are reported. LOSO CV is the proper measure for subject-independent BCI use. The 10-fold CV results show the segment-level upper bound.

Class imbalance and additional metrics. The dataset is imbalanced. For example, Arabic reading includes 872 segments, whereas Arabic listening includes 2125 segments. Therefore, balanced accuracy and macro-F1 are also reported. Class-wise precision, recall, and F1 are kept in the main results table. Balanced accuracy is the mean of per-class recall. Macro-F1 is the unweighted mean of per-class F1. These metrics give equal weight to each class. Thus, they reduce bias toward larger classes. The LOSO CV values are reported in Table 6.

Language-group confound and script/acoustic effects. In this dataset, Arabic and Turkish samples were collected from different participants. Therefore, the language label is partly linked to the participant group. Demographic and session effects may affect the language-detection results. In the reading task, the two languages also use different scripts. Thus, part of the EEG response may reflect visual script processing rather than language identity. In the listening task, both audio files were prepared in the same room with the same setup. However, speaker, prosody, and rhythm differences may still affect the EEG response. These cues may act as acoustic confounds. For these reasons, Case 6 (Turkish reading vs. Turkish listening) is the cleanest mode-detection case. It uses the same participants and the same language. Case 6 gives 98.69% accuracy with 10-fold CV and 72.16% with LOSO CV. This decrease is consistent with the subject-dependent nature of EEG. Case 7 (mode detection on the full dataset) gives the best LOSO accuracy (83.50%). Thus, mode information is the strongest cross-participant signal in this dataset.

Dependence between features. DiagPat builds five transition tables from the same diagonal stream of one signal. Therefore, features within the same table are not fully independent. Features across different tables may also share information. INCA was used to reduce this dependence. INCA is an iterative wrapper method that scores feature subsets with kNN accuracy. It favors features that add new information to the selected set. Strongly redundant features are therefore removed during selection. The final feature count is 861 of 960. The excluded 99 features were the most redundant features. The confusion matrix values for each case are also reported. Accuracy, precision, recall, F1-score, and geometric mean are computed from Figure 2.

By using the introduced DiagPat-driven model, interpretable results have been generated, and a connectome diagram was created for each case. These cortical connectome diagrams clearly demonstrate that the recommended model generates a unique connectome diagram for each case. Furthermore, using these cases, we detected both language and mode (reading or listening) detection.

**Figure 4:**
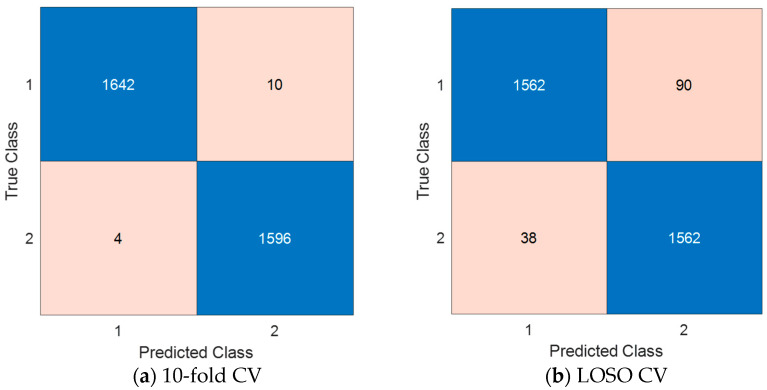
The computed confusion matrices for English and Turkish language classification dataset by deploying the recommended DiagPat-driven XFE model. Here, 1: English, 2: Turkish.

To demonstrate the general classification ability of the recommended model, it was applied to a publicly available dataset containing English and Turkish classes. The comparative results obtained from this dataset are tabulated in Table 7. Moreover, the computed confusion matrices for this dataset have been demonstrated in Figure 4.

Table 7 clearly demonstrates that the presented model achieved 99.57% and 96.06% classification accuracies on the English and Turkish classification EEG dataset with 10-fold CV and LOSO CV respectively. These results highlight that our model outperformed the FGPat-based model in classification performance. Moreover, the FGPat-based model [36] lacks XAI results, whereas the introduced DiagPat-driven model is an XFE model that generates XAI results using the DLob symbolic language. Dogan et al.’s [37] used a directed quantum pattern-based feature engineering model incorporating both tkNN and tSVM, and they extracted the cortical map. In our model, we used DLob and obtained more explainable results, while the DiagPat-driven XFE mode achieved higher classification accuracy than Dogan et al.’s [37] model.

The most important points of this research are discussed below.

Findings of the classification results:

- The model achieved over 91% accuracy across all defined cases, demonstrating robust classification performance.
- Case 1 attained 99.33% accuracy, showing high precision in distinguishing Arabic reading vs. listening.
- Case 2 achieved 93.61% accuracy, highlighting the model’s ability to classify Arabic and Turkish reading tasks.
- Case 3 reached 98.82% accuracy, reflecting effective classification between Arabic reading and Turkish listening.
- Case 4 obtained the highest accuracy at 99.35%, showcasing the model’s strength in distinguishing Arabic listening and Turkish reading.
- Case 5, focusing on listening mode detection, achieved 93.84% accuracy.
- Case 6 reached 98.69% accuracy, indicating high performance in detecting Turkish reading vs. listening.
- Case 7 achieved 98.71% accuracy for language and mode detection across reading and listening tasks.
- Case 8, involving four-class classification, attained 92.35% accuracy, demonstrating reliable multi-class discrimination.
- Case 9 achieved 92.14% accuracy in detecting languages (Arabic vs. Turkish).
- Precision, recall, and F1-scores remained consistent across all cases, further validating the model’s reliability.
- Case 4 had the best F1-score (99.31%), highlighting its balanced performance across all metrics.
- Case 7 exhibited robust recall (98.44%) for mode and language detection tasks.
- Geometric mean scores across all cases exceeded 91%. These results showcase the balanced high classification performances of the presented DiagPat-driven XFE model.
- The highest geometric mean was observed in Case 4, emphasizing its exceptional classification quality.
- The range of classification accuracies (92.14% to 99.35%) indicates consistent and high performance across all cases.
- Multi-class tasks (Case 8) maintained high performance despite increased complexity.
- Classification results confirm the model’s adaptability to different languages, modes, and tasks.
- The DiagPat-driven XFE model demonstrated linear time complexity while achieving high accuracy, ensuring efficiency.
- Comparative results with publicly available datasets validated the model’s generalization ability.
- The recommended model offers linear time complexity, while attaining high classification performance.

Findings of the XFE results:

- Each case produces a unique connectome diagram, indicating that the model captures specific neural activity patterns.
- The frontal regions (FL and FR) show dominant activity across all cases, highlighting their key role in language and mode processing.
- Temporal and parietal regions (TL, TR, PL, PR) also contribute significantly, depending on the case.
- Occipital regions (OL, OR) exhibit lower activation compared to frontal and parietal areas.
- Case 6 demonstrates a balanced activation across regions, reflecting its complexity in reading vs. listening detection.
- Case 8, which includes four classes, shows comprehensive involvement of all cortical regions.
- Histograms confirm the predominance of FL and FR symbols across all cases.
- In Case 1, FL and FR dominate, aligning with its focus on Arabic reading vs. listening.
- Case 2 shows slightly higher TL and TR activity, correlating with reading task distinctions between languages.
- Case 3 involves Arabic reading vs. Turkish listening and reflects balanced FR and TR contributions.
- Case 4 highlights PL and PR contributions for Arabic listening and Turkish reading discrimination.
- Case 5 depicts PL and TL involvement, emphasizing listening mode detection.
- Case 6 has nearly equal contributions from all cortical areas, reflecting its complexity.
- Case 7 balances frontal and temporal activations for language and mode detection.
- Case 8 demonstrates diverse symbol usage due to its multi-class scenario.
- Case 9 highlights consistent FL and FR activity for language detection.
- Information entropy values reveal that the complexity ratio is highest in Case 6.
- The generated DLob sentences successfully reflect cortical dynamics for all cases.
- Cortical connectome diagrams validate the interpretability of the model’s outputs.
- The DLob histograms confirm predictable and distinct symbolic representations for each case.

Advantages:

- The introduced DiagPat-driven XFE model attained over 91% classification accuracy for all cases. The highest accuracy, 99.35%, was achieved in Case 4.
- DLob symbolic language was integrated into the model. This ensures the generation of interpretable outputs and enhances model usability.
- Our model operates with linear time complexity, which demonstrates that the presented DiagPat-driven XFE model is a lightweight model.
- The DiagPat function is based on transition tables. It introduces a novel and efficient feature extraction method, effectively extracting EEG signal characteristics.
- The introduced DiagPat-driven XFE model’s performance was validated on publicly available dataset and our model attained superior classification performance.

## 7. Limitations and Future Directions

**Figure 5:**
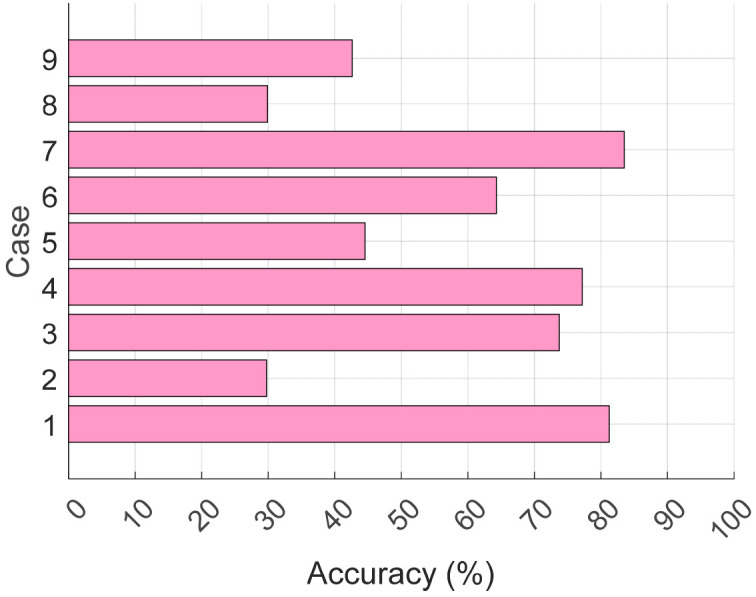
Classification accuracies of the defined cases with LOSO CV. The accuracies range from 29.75% (Case 2) to 83.50% (Case 7). High-performing cases are Case 7 (83.50%), Case 1 (81.25%), Case 4 (77.19%). For Case 8 (multi-class case), the DiagPat-driven model attained 29.89% accuracy.

Limitations:

- The curated EEG dataset is a large dataset, but this dataset contains only two languages. More languages and more modes can be involved.
- The Arabic and Turkish samples were collected from different participants. Therefore, language is partly linked to the participant group. Demographic and session effects may affect the language-detection results.
- Arabic and Turkish use different scripts. Therefore, part of the reading-task signal may reflect visual script processing rather than language identity. Future studies should include same-script language pairs, such as Turkish and English, to control this effect.
- In the listening task, speaker, prosody, and rhythm differed between the two audio files. The recording room and setup were kept the same. However, these acoustic cues may still affect the EEG response. Future studies should use audio matched for length, speaker style, and pace. A single bilingual speaker may also be used when possible.
- The 10-fold CV results are segment-level. The LOSO CV results are subject-level and provide the proper measure for BCI generalization in this dataset. The main claims are therefore reported with both metrics.
- The presented DiagPat-based model reached high classification performance deploying 10-fold CV. However, when using LOSO CV validation technique on the curated dataset, the obtained LOSO CV-based classification accuracies dropped, as showcased in Figure 5.

Future directions:

- We plan to collect a new language detection dataset including more languages.
- New generation subject-specific feature engineering models can be developed. The recommended DiagPat-based XFE model is an EEG segment-specific model. Therefore, it attained higher classification performance with 10-fold CV than LOSO CV.
- In the future, language barriers may disappear. The DiagPat-based EEG system could be the base of a universal brain–computer interface (BCI). It can decode brain activity into a language-free format. People could think in their native language, and the system would translate it into another language in real-time.
- Mental health patients could wear EEG headsets. These headsets would monitor their brain activity and adjust treatments in real time. Therapy could include mindfulness exercises or brain training tailored to each patient.
- In smart homes, EEG systems powered by DiagPat could help people with neurological conditions like epilepsy or ALS. These systems could monitor brain activity, predict episodes, and adapt the environment for safety. Lights could dim, calming sounds could play, or caregivers could be alerted when needed.
- DiagPat technology could allow brain control in harsh environments like space or deep-sea missions. Astronauts or divers could use their brain signals to control robots or monitor their mental state.
- Future classrooms could use EEG systems with DiagPat to enhance learning. The system would monitor students’ focus and stress levels.
- Stroke patients could use DiagPat-driven BCIs to regain speech and communication skills.

## 8. Conclusions

In this research, a new EEG language and mode classification model was presented and this model is named the DiagPat-driven XFE model. The introduced DiagPat-driven XFE model achieved over 91% classification accuracy across all defined nine cases, with the highest accuracy of 99.35% observed in Case 4. This case (Case 4) consists of Arabic listening and Turkish reading tasks. These results validate the introduced DiagPat-driven model’s high classification performance. The 10-fold CV results are segment-level. Subject-independent performance was also tested with LOSO CV. The LOSO accuracies range from 29.75% to 83.50%. The best LOSO result was obtained in Case 7 (mode detection, 83.50%). The lowest LOSO result was obtained in Case 2 (Arabic vs. Turkish reading, 29.75%). This gap shows that part of the 10-fold gain comes from segment-level information. In this dataset, mode-related information generalizes better across participants than language-related information.

By integrated the DLob method into to this model, the cortical connectome diagrams were generated for each case using the generated DLob sentences. The DLob sentences summarize the brain activity for each case. Moreover, the generated DLob sentences showcased the dominance of the frontal lobe for the defined cases’ classification.

The DiagPat-driven XFE model demonstrated adaptability across various scenarios, including language detection, mode detection, and multi-class classification tasks. Its application to publicly available datasets further validated its generalization capabilities, achieving a classification accuracy of 99.57% with 10-fold CV and 96.06% with LOSO CV for English and Turkish EEG signals.

The introduced DiagPat-driven model combines high accuracy, efficient processing, and explainable outputs. In this aspect, the recommended model contributes to feature engineering, brain-based language research and neuroscience.

## Figures and Tables

**Table 1:** Summary of related EEG-based language and speech studies.

Ref.	Method	Dataset/Task	Language(s)	Subjects	Accuracy (%)
[18]	ChannelPat + Lobish + INCA + tkNN	20 daily life sentences	Arabic, Turkish	75	98.59
[19]	DSBP + IMCMV	20 sentences, display + listening	English, Turkish	40	98.81/98.19
[20]	Correntropy SD + Riemannian	Kara One; imagined speech	English	8	90.25
[21]	TesPat + NCA	Three sentence datasets	Turkish, English	n.r.	>97 CV; >91 LOSO
[22]	CNNeeg1-1	Imagined vowels (BD1, BD2)	Spanish	15/50	65.62/85.66
[23]	ResNet18 + 2GRU	Silent speech, 9 words	Russian	268	84.50
[24]	MC-CNN	Imagined nouns vs. verbs	English	n.r.	78.50 (LOSO)
[25]	DWT + SVM	UNER imagined speech	Spanish	15	79.78/26.75
[26]	CNN	Speech perception	Dutch, Frisian	26	71.00
[27]	PSD+SVM, EEGNet	Spoken words	Spanish	28	EEGNet best in 3/4 cases
Ours	DiagPat + INCA + tkNN + DLob	Reading + listening (9 cases)	Arabic, Turkish	346	92.14–99.35 (10-fold); 29.75–83.50 (LOSO)

n.r.: not reported. CV: cross-validation. LOSO: leave-one-subject-out.

**Table 2:** Attributes of the curated EEG signal dataset.

No.	Class	Mode	Number of EEG Segments	Number of Participants	Age
1	Arabic	Reading	872	49 (40 M + 9 F)	19 to 51
2	Arabic	Listening	2125	49 (43 M + 6 F)	19 to 48
3	Turkish	Reading	1273	137 (118 M + 19 F)	18 to 69
4	Turkish	Listening	2094	111 (105 M + 6 F)	18 to 47
Total/Overall			6364	346	18 to 69

**Table 3:** The parameters used of the introduced DiagPat-driven XFE model.

Phase	Method	Parameters
Feature extraction	DiagPat	Size of the matrix: 14 × 14, The used values for feature extraction: Diagonal values, Feature extraction method: Transition table-based feature extraction, Number of the features: 960
Feature selection	INCA	Range of the iteration: from 100 to 960, Number of the selected feature vector: 861, Classification value calculator: kNN with 10-fold CV, Selection method: Greedy.
Classification	tkNN	Distances: City block, Euclidean, Cosine, Weight: Inverse, Equal, k values: from 1 to 5, Number of classifier-wise outputs: 30, Voted outcomes generation method: IMV, Sorting factor of the IMV: Descending order based on classification accuracy, Range of the IMV’s loop: from 3 to 30, Majority function of the IMV: Mode, Number of the voted outcomes: 28, Number of the total outcomes: 58, Selection the best outcome: Greedy.
XAI	DLob	Number of symbols: 8, Symbols: {FL, FR, TL, TR, PR, PL, OL, OR}, Statistical analysis: Histogram extraction, information entropy computation, transition table computation.

**Table 4:** The classification results (%) of the defined cases employing the introduced DiagPat-driven XFE model.

Case	Accuracy	Precision	Recall	F1-Score	Geometric Mean
1	99.33	99.36	99.02	99.19	99.02
2	93.61	93.56	93.17	93.36	93.14
3	98.82	98.63	98.53	98.58	98.53
4	**99.35**	**99.39**	**99.23**	**99.31**	**99.23**
5	93.84	93.90	93.82	93.86	93.81
6	98.69	98.55	98.67	98.61	98.67
7	98.71	98.67	98.44	98.56	98.44
8	92.35	92.01	91.76	91.88	91.72
9	92.14	92.80	91.64	92.22	92.18

**Table 5:** The computed information entropies for each case.

Case	1	2	3	4	5	6	7	8	9
Ent.	2.62	2.59	2.67	2.59	2.35	2.68	2.65	2.50	2.59
Rat. (%)	87.33	86.33	89	86.33	78.33	89.33	88.33	83.33	86.33

Ent.: Information entropy, Rat.: Complexity ratio = (Information entropy)/(Maximum entropy). Herein, maximum entropy is equal to 3 (=log_2_(8)) since we have used 8 DLob symbols.

**Table 6:** LOSO CV results with balanced accuracy and macro-F1.

Case	Accuracy (%)	Balanced Acc. (%)	Macro-F1 (%)	Note
1	81.25	74.10	72.55	Arabic reading vs. listening
2	29.75	30.42	28.86	Arabic vs. Turkish reading
3	69.30	62.81	60.94	Arabic reading vs. Turkish listening
4	77.19	70.45	68.77	Arabic listening vs. Turkish reading
5	56.34	54.10	52.68	Arabic vs. Turkish listening
6	72.16	67.92	66.40	Turkish reading vs. listening (within-language)
7	83.50	81.75	80.62	Mode detection (reading vs. listening)
8	29.89	30.08	28.45	Four-class
9	63.94	59.31	57.82	Language detection (Arabic vs. Turkish)

**Table 7:** The comparative results about to language detection using English and Turkish languages.

Research	Model	XAI	Accuracy (%)
Kirik et al. [36]	Feynman graph pattern-based language detection model	No	10-fold CV: 99.38, LOSO CV: 92.47
Dogan et al. [37]	Directed quantum patter-based model	Yes	LOSO CV: 95.68
Our model	DiagPat-driven XFE	Yes	10-fold CV: 99.45, LOSO CV: 96.06

LOSO: Leave-One Subject-Out.

## Data Availability

Authors do not have permission to share data.

## Supplementary Materials

The following supporting information can be downloaded at: https://www.mdpi.com/article/10.3390/s26113363/s1, Table S1: The DLob sentences generated by the proposed DiagPat-based model; Figure S1: The histograms of the extracted symbols per the cases; Algorithm S1: The MATLAB code of the presented DiagPat; Algorithm S2: MATLAB code of the DLob-based XAI results generation function.
