# Supplementary material for: DiagPat: An Explainable Language Detection Model Using EEG Signals
Source: Sensors (Basel). 2026 May 26;26(11):3363. doi: 10.3390/s26113363 (PMC13258914; doi:10.3390/s26113363)
Supplement: Supplementary file 1 [file sensors-26-03363-s001.zip › sensors-4305761-supplementary.pdf]

The generated DLob sentences have been demonstrated in Table S1.

Table S1. The DLob sentences generated by the proposed DiagPat-based model.

C	DLob sentence
1	FRFRPRORFRPLFRFLFLFRFLTRFRFLFRFRTRTRFLPROLPLFRFRPRTRFLFRFLF<br>ROLTLPRPROLORFLFRFLFLORORFLFLFLTLFLFLPRFLFRPLTRORFLFLFRFRF<br>LFLFRFRFRFRTLOLFLFRTLFLTLFLPRFRFRFRORPLTRPRTLOLTLORPLTLFRTL<br>FRFRFRFRPLOLFRFLOLOLFLOLPROLPLPLFRFRFRFRFLFLFLFRORPRFRFLPR<br>TRFLFRPLFROLOLFLFLFLFLFLFLFRFRFRPLOLTLTLFLORORFROLFRFROLFL<br>FLFLFLFLFRFROLFRFRFLTRFROROLFLFLFLFRFRFRFLFLFRFLFROLFRTRFRFL<br>FRFRTLPLFRPLTRFLOLFRPLFRFRFRFRFLFRPRTLFRFRFLTLTLFLFLFLFRFRFR<br>FRFRFLFLFLFLFRPRTROLFLFLOLPLTRPRFLFLPLTRFLPRTLFRPLFROROLPLF<br>RTRPLPRFLPRFRFLFLFRPRFROROLFLPRFLFLFRPRFRFRPRFLFRFLPRFRFLFL<br>FRFLTRFRFLPLOLFRFRFRORFRPRTLPLFRTRORFRFRFLORFRFRFRFRFRFLFRF<br>RFLFRFRFRFRTRFLFRFRFRFRORTRFLFRPRFRORFRFRFLPLTLORFRFLFLFRFR<br>OLTLTRFRFLFLFRTLFRFLTLFLFLOLOLPRFRPLPLPLFLFRPLFRPRFRFLFLPLO<br>RTLFRPRPLFRORFRFLOLTRORTLFRFRFRPRFLFLFLTLTLOLFLOLPLPRFRPLF<br>LTLFLFRFLFRPLFLOLFRTLPLPRFLFLFLFROLOLORFLFLPRPRFLPRFLFLFRFR<br>OLFLFLPRFLFLOLTLFLPLFLFLFLFLOLFLFLTLTRFRFRPLFLTLPLPLPRFRPLTR<br>FLFLFROLFLFRFRORTRTRFRPLFRFLPRFLPLFRPLFRFLFRPLFRFLPRTLOLFRF<br>RFRFLFRTLFRFROLFLPLTLORTLTRPLFROLFLPLTRFLFLFLTLPLFLFLOLFLOL<br>FRFLFLFRPROLFRTLFLPLFRFRFLFRFLFRPLFLPRPLFRFLFLFRFLFRFRTLFLFLF<br>LTRFRFRPRFRTRFLTRFROLFRFLFLOLPRFRFLTLFLFRFLPRTLTLPLFLFRTLFL<br>TLFLFLFLFLFLOLFRPLPRFRFRTLFLOLFRPLFLFLFLFLFLFLPRFRFRORTLFRFL<br>FRPRFLFLFLFROLPLFLPRTLOLOLFRORPRFLFLTLFRFLORFRFLFRFRTRPLTLT<br>LORORTLFRTRFRTRFLPLFLFLORFLFLTLORFRTRPRPRFLTRFLPLPLTRFRFRF<br>LFLOLFLFL
2	PRPRFRFRFLFLFLFRFRFRFRFLFRFROLOLPROLOLFLOLFRFLFLTRPRPLFLFRF<br>LFLFLPLTRTLFLFLOLTLFLFRFRTLOLFRFRFLFLTLPLOROLPLFLFRFLPLPLFR<br>FRPLFROROLOLPRFRFLFLFLPLTLTLFRFRFRFLFLORORFRFROLPLFRPLFLFLF

	RPLFRFLTRTRFRFRTRPRFLFLFLFLFRPRFLFLPLOLPLFRPLFLFRFRFRFLFRFRF<br>ROLFLTLOLFLFRFLORFRFLTLFRFRPRFRFRFLFRFRPLPLFRFRFLFRFLOLOLO<br>RFLFLPLFRTLPLFRPLFLFRFRFRFLFLFLPLFLFRPLPLPLFLPRFRFRFRFRTLFRFL<br>PLTLTRFRFLTLFROROROPLFRFRFLFRPLORFLFRFLPLFRPLORFRFLFRFRPRFRT<br>RPLOLPLFLFRFRFRFRFLFLPRTRFLFROLFLPLFLTRFLOLTLTLTLFLFRTRFRFL<br>FLFLOROLTRPLFLOLPLFLFLFRFLPLFRTLTLFLFLFRFLFRFLOROROFLFLPRORP<br>RPR"
3	FLTLFLFLFRFROROROPRORPROLTRTRPLFRFRFRFLFLTLFLOLORFRFRPRPRTL<br>TLFRPRPLTLFRFRPRFRFLFLOLOLPLOLTRPLPLFRPLTLFRFLOLPLFLTROLOL<br>FLTLFLFLFLFLTRTRFRPRFLFLPLPRFRPLFLTRTLPLFRFRTRPRFLFLFRFROLTL<br>TRFLFRFRPRTRFRFROLFROLTLFLFRFLFLFRPLOLORFRTRFRFLPLFRTLFLPLF<br>RORFLFLFRPLORTLFRPLFRFRFLPRFRPLPLFRPLFLFRFLPLTRFLFROLFRFLFL<br>FLPLFRFRFRFLFLPLFLFRFLTRPRFLFLOROLTLFRFRPLFRFLFRFRFLFLOLPRPR<br>FRORPRPRFLFLTRFLFLFLFRFRFRFLFRFLPROLFRFLFRFRTLFLPLTRFRFLFRFL<br>OLFRFRPRPRFRFLPLOLTLFROROROLFLOROLTLTLFLFLFRPRTLPLFRPLFLFR<br>FRFLFRFRFRTLFLFLFLFLTRFLFLFLORPLFRTLFRFRORTROLPLFROLFLFLTLF<br>LFLFRFLORTRORFLFRPLFRPLFLFLFLTLFLPRTRFLTLFLFLTLORFLFLOLFRFR<br>FRFLFLFRFRFRFRFRFLFLFRFRPLTLOLFRFLFLFLOLTLTLFLFLTLFROLPRPLFL<br>FL
4	OLORFLFLFLFLFRFRTROLTLPLPLOLFRFRFLFLFRFRFLOLFRFLFLFLFLFLFLF<br>RTLTLTRTRTLFLFLFROROROFRFRTRFRTRPROLPRPRORPRPRFRFRFRFLFLFLP<br>LPLFRFRPRPRFRFRORTLFLFLFRFLFRPLOLTRFLFLOLOLFRFRFLFRFLFLTLFL<br>FLFRPLOLFLFRPLTLTLTRPRTRTRPLFRFLFRFLFRTRFLFLFRFLFRFROLFRFLFL<br>FLFLFLFLTRORFRTRFLFLFRFRFLFRFLFLFRFROLFRORPROLTLPRPLTRPRPLF<br>RFRPLOLFLFLFLFRORFRTLTRFRPLPLFROLFRPRFRFRFLFRFRPLFLFRFLFROL<br>OLFRTRFLFLFLFLFRFLPLFROROROFRFRFLTLFLTLFRFLFRFRFRFLTRTLFLFLPR<br>TRTLFLFRTRTRFRFLFLOROLTRFRFLFRFLFLFLFRFRFLTLOLPRFRFRFRORFRP<br>LFLFRFR

5	FRFRFRFRFLFLFRFRFRFRPLPLFLFROROFLFLTLFLFLFRPLOLFLTLOLORFRF<br>RFLFLFLFLFRFRFLFLFLFLTLPLFRFLFRFLFRFLFLFLTRTRFRFLFRFRFRFLTLTL<br>TLFLFRFRPRORPLFLFLFLFRFROLFRFLFLFLFLFLPLFLORFRFRFRFRFLPLFLFR<br>PRFRPRTRFLFLFLORFLTRFRFLFLFRTRFRFLFLTLTLFLFLFRFRFLFRFRFRFLOR<br>TRFRFLFLFRFRFLPRTLFRPRPRPLFLFRFLFLOLPLFROLFRFRFRFLFLTLFROLF<br>RFLFLFRFLFRFLFLOROLPLFLFRFRPLPLTRFRFRPRFLOLPLFLFLFLFRTLFLOR<br>ORFRFLFLTLFLFRFRFRFRFLPLTLFLFRFRPRORPLPLPRFRFLORORFLFLORPRF<br>LFLFRFRFRFLTRPRPLFLFLFLFRFLFRTRPRFRFRPRFRTLFRFLFRFRFRTLFRFRF<br>LPRFLFRPLFRFRFRFLFRFRFRFLTLFLFLFLTLTLFLFRFRFRFROLOLFLFLFLTRF<br>LFRFRFRFRFRFLFRFLTRFLPLFLFRFRFRTLFLTLFRTRFLPLFRFROLFRFRFLFLF<br>LFLFLFRFRPLFRFRFRFRFLFRFLPLFRFRTRFLFLFLFRFRORFLTRFRPLFRTRPLF<br>ROLFLFLFROR
6	OLOLPRPRFLFLFLFLFRFRTRTLFRFRPLOLORORFLTLTRPRFRFRFRFRPLTLFL<br>FLFRFLFLFRFLPLFLFLTRTRFLFLFRFRFLFLOLTRFLPRORPRFRFLPRTRORORT<br>LTLFLFLFRFLPRPLFRFRFLFLTRPLFRFRFLTLOLPLOLFLFRFLFLFLFLFLFRPLO<br>LPRFLFLFRFRFLFRTLOLTLTLFLFLPLFLFLFLPLTLFLFLPLORFRPLTLFLPLTRF<br>LFRFRFRFRFLTLFLOROLTRTRFLFLFRTRPRPRPLFRFRPLTRFRFLFLTLPLFRFR<br>FLFROLOLPRFLTLTRTLFRTRFRFLFRPRTRPLFRFLFRFRFRPRORFLPLFRFROR<br>OLOLORFLFLFRFRFRFLFLTLPRFRFRPRTLFLFLFLFLFRFLFRFRFRFLFLFRFRFR<br>TRFLFLFLTLFLPLTRPRFLOLFLFLFRFLFRPRFLTLFRFRPLPLFRFRTLTLTLFLTR<br>FROLFLFLFLTLOLPRTLFRTLFLTLFRPLFLPRFRFLFLOLOLTLFLFLFLFRFRPLF<br>RFLFRTRTLPLPRFROLPLOLFRFRFLPRFLPLFLPLFRFLFLPROLFLFLPLFRFRFLF<br>LFLPLFRFLFRPLPLFLFRFRTROLFLORPLFRFLFROLTLFLPLFRFLTRPLFLTRFLP<br>RFRPRFLFLFRFLFLFRPROLORPLFRFRTLFRFLOLTLTRTLFRTLFLTROLTLFLFL<br>OLFLTRFRFLFLFLOLPLORPLOLPRORPLOLOLFLOLPLPLFRFRFRTRFRTROLF<br>LPLTLFRFRPLORPLFRPRFLPLPRFRPLFLFLFLPRFRFRPLORPLFRPLTLFLPLFLP<br>LPLFRTLTRFLFRPRTLFRPLORFLFLFLFRTLTRTLPRFLFRTROLFRORFRTRFLFL<br>FLFLFLFROLFRFLFR

7	FLTLFRFRFRFRPRPLFLFLFLFLTRPROROFLFLTRPRFLPRTLPLFLFRPLPLFRF<br>RFLFLFRFRFRFRFRPLTLFROLOLFLFLFLFLFLFLTRTRORPLFLTLFLFLFLFLFRF<br>RFRFLFLTLFRFLFLFLOROROPRPLOLFLFRFLFLFRFRFLFLFRFRFLFLFRORFR<br>PLOLFRFROLTLFLFLFLTRFRPRFRTLTLFRFRFLOLFRPLFRFRFLFLTLOROLPRP<br>LTLFLFRFLFLOLORFLFRFRFLTRTLFLFLFLTLFLFRTROLPRPRPLTRFRFRFLFR<br>FLOLPRFLFRFLPLOROLPLFRFRFRFRTLTLFRPLFRFLFRFRFRFRTLPLOROLFRF<br>RFLFLFLFLTLFLPROLPRORPLFRFRFLOLTRPLTRFRFLFRFLPRTRFRFRTRFLFL<br>TLORTLPLFRFRPRFLFLFRFLOROLFRTLFLFRFLFLFRFLFRFROLTLFRTRFRFRP<br>RFRPRTRTRPLFLFLTLFRFLOLTLFRORFRFRTRFLTRPLOLFRPRTLFLFLFRFRFR<br>TRTRFROLPRFRFRFRFLFRPLTLFLFLPRPRFRFRORTLFRFROLTLTRPL
8	FLFRPLTLFRFLFLFLTRTRPLPLTLTLOLOROLTRFRFLFLTRFRFRFLFLFRFRFRF<br>RPRORTLFLFRFLOLTLTLFLFRFRFLFLPLFRFLFRFRFRFLFLPLTRFLFLTRPRFRF<br>LTLFLOLPLFRFRFRFRFLTRFRFRFLFLFLFLFRFRFLFLPLFRPLOROLOLORORP<br>RPRFLFLPRFRFLFLFRFLFRFRPLPLTLFRFRFRFLFLPLFLFRFROROFRFLFLFRT<br>LFRFRFLFLFLFRFRTLTLPRPRFLFRFRFLFRTRPRFRFRTLTLFLTLPLFRPRFLFLF<br>RFRFLPRFRFRFRFLPLOLFRFROLFLTLFRPLOLFLFRFLFLPLTLFLOLTLOLFLFR<br>FLFLFRFRFRFROROLFLFLTLFLTLORTLTRFLFRFRFLFLFRFLPLOLFRFLFLFRP<br>RFLOLORORFRFRPRFLFLPLPLFRPROLFLFRFRFRFLFLFLFLPRFLFRFLFRFLFL<br>FLFRFR
9	FLFLPLPLORFLFRFRFRFLTLTLFRORFRFLFLFRFRFROLFRFLFRFRFLFLFRFRFR<br>OLPLOLORTLFLPLTLFRFRPRFRPLTLFRFRFLFLPRPRFRFRFRFRPLTLFLTLPRT<br>RFRFRFLFLFLFLTRFRFLFRFLORTLOLFLFLFLFLTRPLFLTLPLFLTRFLFLFLPLF<br>LTRTRFLFROROPRFLFLFRFRFRFLFLFLOLFRFLFRFRFLFLFRPRTLFLFLPROL<br>ORTLPLTRTRFLFLFRFLFLFLFLPLPRFRFLFRFLPLFLTRPLFROLFLPLOLTLPLO<br>RFLFRFROLOLORPRFLPLFRFRFRFLFLFLORFROROLFLFLOLTLOROLORORFR<br>FRFRFROLPLORFRORFRORFLFROLPLFLFLTLFRFRFLFRPLFLFRFRFRPLFRFLF<br>LFLFLFRFRFLFRFRFLORFROROROFRFLFRFLFLFRFLFROLFRFRFRPLFLPRFL<br>PLORFRPRFRTRFRFRPRFRFRFRFRFLPLTRFLFRFLFLFRFRFROLPRPRFRFLTRF

	LPLFLFRORFLORFRFLFRPRFRPRFLPLFLTRFLPRFRPLORPRFLFLTRPRFRTRPLFRPRFRPROLFRFRFLFLFRPLTLFLFLORFLPLFRFLPRORFLOLTRFRPRORFLFLPLORFLFLPLFRFRPRFROLFRFRPLFRFRFLFLOLFLFRFLTLFLPLTLORFLPLPLFRPRFRTLFLFRPLFRPLTLFRFLFLTLPRFLFRFRFLPRFLORFLPRFLTLPLTLFLPRFRFLFLFLPRFLFLPLFLFLFRPLPLFLORTLFLORTLFRFRFRFLFRTLFLFLORFLTLFLOLFLTRFRPLFRFLFRFRFRTRFRFLTLFRPLFLOLFRFRFRFRPLFLFRTLOLFLOLFRFRORFLFRFLORORFLFLFLFRFLFLORFLTLTLOLFLFLORPL

C: Case

The histograms of the used symbols per the defined cases have been depicted in Figure S1.

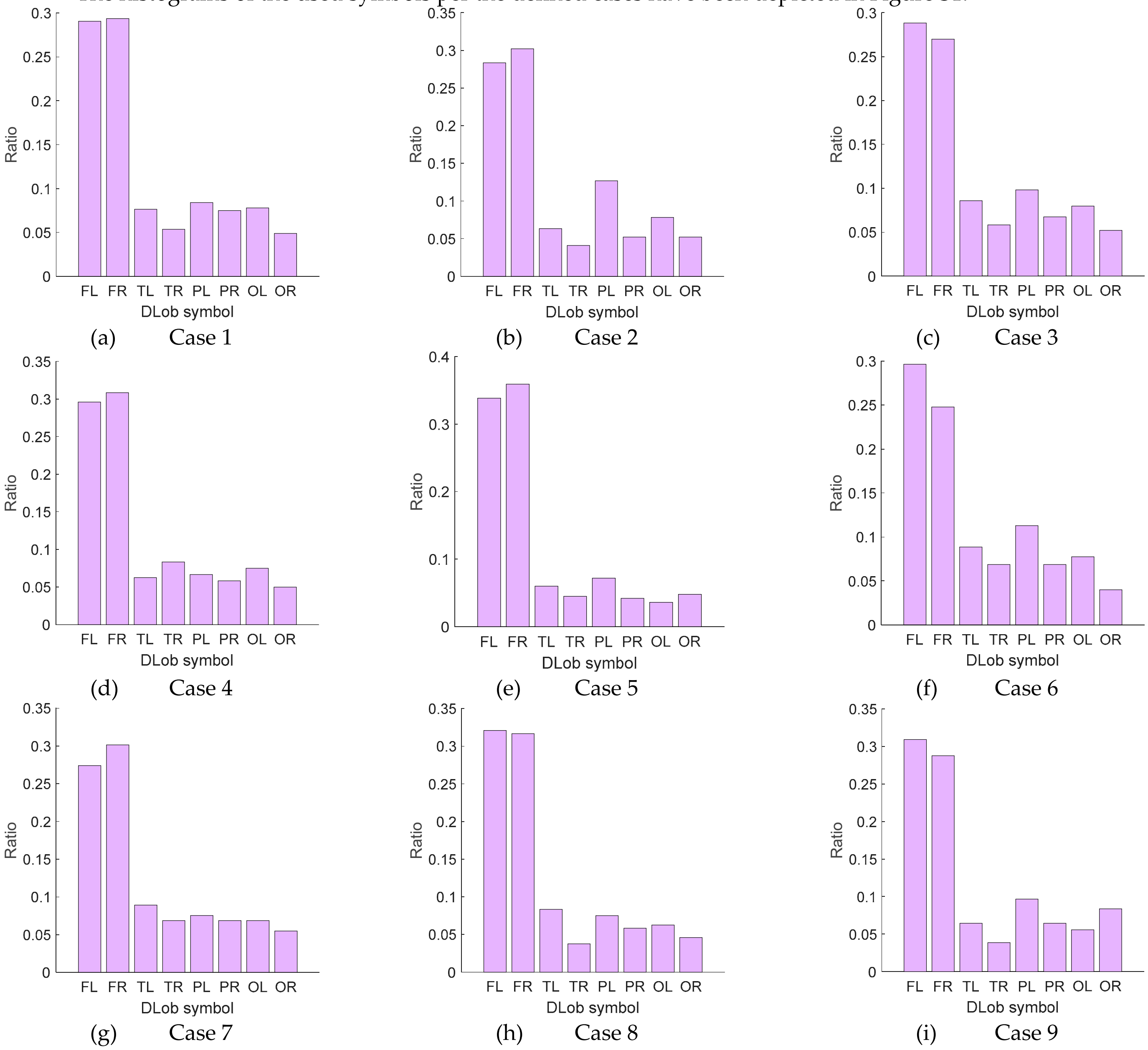

Figure S1. The histograms of the extracted symbols per the cases.

In the Algorithm S1, the MATLAB code of the presented DiagPat-based feature extraction function.
Algorithm S1: The MATLAB code of the presented DiagPat.

```
function vek = DiagPat(signal)
  % Function DiagPat: Extracts a feature vector from the given signal using diagonal patterns.
  % Input:
  %   signal - Input signal (matrix format).
  % Output:
  %   vek - Extracted feature vector.

  [rows, cols] = size(signal);  % Get dimensions of the input signal.
  channel = min([rows, cols]); % Determine the number of channels.
  length = max([rows, cols]);  % Determine the longer dimension of the signal.
  tt = zeros(channel, channel, 5); % Initialize a 3D transition table with zeros.

  % Ensure the signal is in the correct orientation.
  if length == cols
    signal = signal';  % Transpose the signal if necessary.
  end

  % Iterate over the signal to extract patterns.
  for i = 1:length - channel + 1
    % Extract a segment of the signal for analysis.
    segment = signal(i:i+channel-1, :);

    % Sort each row of the segment in descending order and store indices.
    for j = 1:channel
      row = segment(j, :);          % Select a row from the segment.
      [~, idx] = sort(row, 'descend'); % Sort row in descending order.
      sortedSegment(j, :) = idx;    % Store the sorted indices.
    end

    % Extract diagonal and reverse diagonal values.
    for j = 1:channel
      d1(j) = sortedSegment(j, j);            % Main diagonal.
      d2(j) = sortedSegment(j, channel+1-j);  % Reverse diagonal.
    end

    % Update the transition table based on the extracted indices.
    for j = 1:channel-1
      tt(d1(j), d1(j+1), 1) = tt(d1(j), d1(j+1), 1) + 1; % Main diagonal transitions.
      tt(d2(j), d2(j+1), 2) = tt(d2(j), d2(j+1), 2) + 1; % Reverse diagonal transitions.
      tt(d1(j), d2(j+1), 3) = tt(d1(j), d2(j+1), 3) + 1; % Mixed diagonal transitions.
      tt(d2(j), d1(j+1), 4) = tt(d2(j), d1(j+1), 4) + 1; % Reverse mixed transitions.
    end

    % Update the transition table for diagonal self-mapping.
    for j = 1:channel
      tt(d1(j), d2(j), 5) = tt(d1(j), d2(j), 5) + 1; % Diagonal mappings.
```

```
        end
      end

      % Flatten the transition table into a feature vector.
      vek = tt(:)';
    end
```

In Algorithm S2, the MATLAB code of the XAI results generation with DLob for the used brain cap.

Algorithm S2. MATLAB code of the DLob-based XAI results generation function.

```
clc;
clear all;
close all;

% Load all .mat files in the directory. These mat files contain the results
% of the cases
files = dir('*.mat'); % Get the list of .mat files in the current directory
for t = 1:length(files) % Loop through each file
  symbolicSequence = []; % Initialize symbolic sequence for the current file
  load(files(t).name);   % Load the .mat file containing data variables

  % Define DLob cortical region labels and their corresponding symbolic mappings
  lobish = ["FL", "FL", "FL", "FL", "TL", "PL", "OL", "OR", "PR", "TR", "FR", "FR", "FR", "FR"];
  % 'lobish' represents labels for different cortical areas such as Frontal Left (FL), Temporal Left
(TL), etc.
  trt = {[1], [1], [1], [1], [3], [5], [7], [8], [6], [4], [2], [2], [2], [2]};
  % 'trt' provides a mapping of channel indices to specific symbols (used for transitions)

  % Initialize histograms for channels and symbols
  histo = zeros(1, 14); % Histogram to count the frequency of each EEG channel
  histo_s = zeros(1, 8); % Histogram to count the frequency of each symbol
  c = 1; % Counter for storing channel indices
  g = 1; % Counter for storing symbols

  % Iterate over the data to extract DLob symbols and construct sequences
  for i = 1:size(son, 2) - 1 % Loop through the data points in 'son' (data array)
    channelIndex = ind(i); % Get the channel index for the current data point
    % Map the channel index to the corresponding cortical regions
    t2 = mod(floor((channelIndex - 1) / 14^1), 14) + 1; % First channel
    t3 = mod(floor((channelIndex - 1) / 14^0), 14) + 1; % Second channel

    % Map channel indices to their corresponding symbols
    for j = 1:length(trt{t2})
      sembol(g) = trt{t2}(j); % Append symbols for the first channel
      g = g + 1;
    end
    for j = 1:length(trt{t3})
      sembol(g) = trt{t3}(j); % Append symbols for the second channel
      g = g + 1;
    end
```

```
        % Construct symbolic sequences from the cortical labels
        kanal(c) = t2; % Store the first channel index
        kanal(c + 1) = t3; % Store the second channel index
        symbolicSequence = strcat(symbolicSequence, lobish(kanal(c))); % Append first channel label
        symbolicSequence = strcat(symbolicSequence, lobish(kanal(c + 1))); % Append second
channel label
        c = c + 2; % Update the counter for the next pair of channels
    end

    % Compute histograms for channels and symbols
    for i = 1:length(kanal)
        histo(kanal(i)) = histo(kanal(i)) + 1; % Increment channel frequency
    end
    for i = 1:length(sembol)
        histo_s(sembol(i)) = histo_s(sembol(i)) + 1; % Increment symbol frequency
    end

    % Create a transition matrix for the symbols
    tt = zeros(8); % Initialize an 8x8 transition matrix
    for i = 1:length(sembol) - 1
        tt(sembol(i), sembol(i + 1)) = tt(sembol(i), sembol(i + 1)) + 1; % Update transitions
    end

    % Compute information entropy based on symbol probabilities
    entropy = 0; % Initialize entropy
    for i = 1:8
        pr = histo_s(i) / sum(histo_s); % Calculate the probability of each symbol
        if pr > 0
            entropy = entropy + pr * log2(pr); % Update entropy if probability > 0
        end
    end
    entropy = -entropy; % Convert to positive entropy value

    % Plot normalized symbol histogram
    cizdir(histo_s / sum(histo_s)); % Plot histogram of symbol probabilities

    % Plot EEG channel histogram
    figure;
    bar(histo); % Plot the frequency of EEG channels
    title('Channel Frequency Histogram');

    % Circle plot for the transition matrix
    weights = tt; % Use the transition matrix as weights
    nodeNames = {'FL', 'FR', 'TL', 'TR', 'PL', 'PR', 'OL', 'OR'}; % Define node labels
    figure;
    G = digraph(weights); % Create a directed graph
    h = plot(G, 'Layout', 'circle', 'EdgeLabel', G.Edges.Weight); % Plot graph as a circle
    title('Cortical Transition Network (Circle Plot)');
    labelnode(h, 1:numel(nodeNames), nodeNames); % Label nodes with cortical region names
```

```
    h.NodeColor = 'r'; % Set node color
    h.LineWidth = 1.5; % Set edge line width
    h.MarkerSize = 7; % Set node marker size
    h.EdgeAlpha = 0.7; % Set edge transparency

    % Save the symbolic sequence for the current file
    katar{t} = symbolicSequence; % Store the symbolic sequence in a cell array
    clear sembol kanal son symbolicSequence; % Clear temporary variables for the next iteration
end
```
